# Testicular seminoma after the complete remission of extragonadal yolk sac tumor : a case report

**DOI:** 10.1186/1471-2490-4-13

**Published:** 2004-11-16

**Authors:** Isao Kuroda, Munehisa Ueno, Tomoko Mitsuhashi, Ken Nakagawa, Hitoshi Yanaihara, Takuji Tsukamoto, Nobuhiro Deguchi

**Affiliations:** 1 Department of Urology, Saitama Medical School, Moroyama, Saitama, Japan; 2 Department of Pathology, Saitama Medical School, Moroyama, Saitama, Japan; 3 Department of Urology, Keio University, School of Medicine, Shinanomachi, Shinjyuku, Tokyo, Japan

## Abstract

**Background:**

Between 2% and 5% of malignant germ-cell tumors in men arise at extragonadal sites. Of extragonadal germ cell tumors, testicular carcinoma in situ (CIS) are present in 31–42% of cases, and CIS are reported to have low sensitivity to chemotherapy in spite of the various morphology and to have a high likelihood of developing into testicular tumors. A testicular biopsy may thus be highly advisable when evaluating an extragonadal germ cell tumor.

**Case presentation:**

A 36-year-old man was diagnosed as having an extragonadal non-seminomatous germ cell tumor, that was treated by cisplatin-based chemotherapy, leading to a complete remission. In the meantime, testicular tumors were not detected by means of ultrasonography. About 4 years later, a right testicular tumor was found, and orchiectomy was carried out. Microscopically, the tumor was composed of seminoma.

**Conclusions:**

We herein report a case of metachronous occurrence of an extragonadal and gonadal germ cell tumor. In the evaluation of an extragonadal germ cell tumor, a histological examination should be included since ultrasonography is not sufficient to detect CIS or minute lesions of the testis.

## Background

Between 2% and 5% of malignant germ-cell tumors in men arise at extragonadal sites [1]. Cytogenetically most extragonadal germ-cell tumors (EGGCTs) i.e., the seminomas and non-seminomas, are similar to their testicular counterparts [2,3]. But there are CIS in 31–42% of EGGCT patients' testes [4]. Ultrastructural studies indicate that CIS originate from rather primitive cells and can develop into different categories of germ cell carcinomas. Furthermore, since CIS are reported to respond poorly to chemotherapies, a metachronous development of testicular cancer will possibly occur, in spite of the various morphologies of testicular cancer [5,6]. In the present case, the etiology of a metachronous appearance of EGGCT and testicular cancer is discussed.

## Case presentation

**Figure 1:**
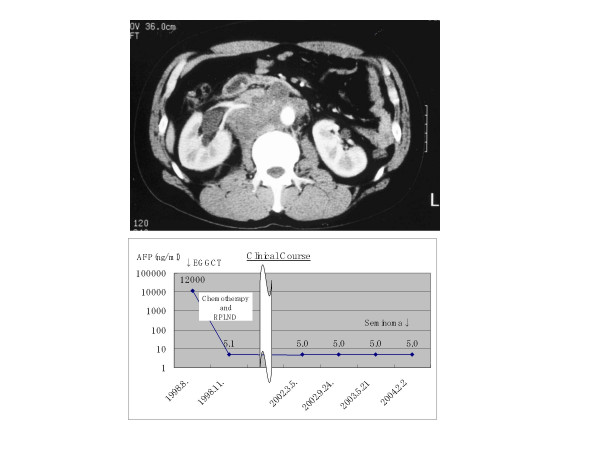
CT shows a retroperitoneal bulky mass. (B) The clinical course from the treatment of EGGCT to that of seminoma.

**Figure 2:**
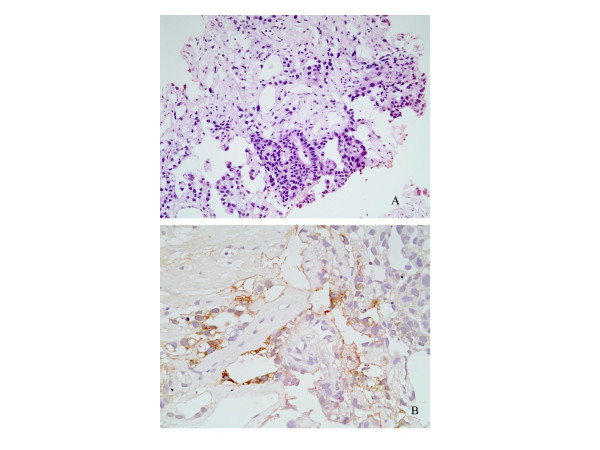
Photomicrograph of extragonadal non-seminomatous germ cell tumor with a focus of yolk sac tumor. A: H&E section shows characteristic structures suggestive of Schiller-Duval bodies (×200). B: Alpha-fetoprotein immunohistochemical stain is focally positive at the above area (×400).

**Figure 3:**
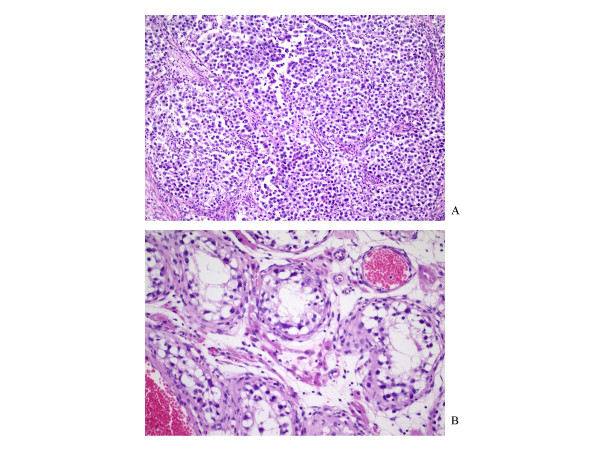
Photomicrograph of classic seminoma of the testis. A: H&E section shows compact nests of large tumor cells are separated by thin fibrous septa infiltrated by lymphocytes. B: Intratubular germ cell neoplasia in H&E section (×100). A row of atypical germ cells with clear cytoplasm is seen against a thickened basement membrane. No spermatogenesis is occurring in this tubule (×200).

A 36-year-old man was admitted to our hospital with the chief complaint of right-sided scrotal enlargement. He had previously received treatment for an extragonadal germ cell tumor. At the age of 32, he presented with lumbago. CT showed a retroperitoneal tumor (Figure 1), and a transabdominal needle biopsy was carried out. Microscopically, the tumor was composed of a yolk sac tumor (Figure 2A,2B). We performed two courses of systemic chemotherapy using bleomycin, etoposide, and cisplatin, leading to a partial response. As the tumor size was not seemed to decrease after the two courses of the chemotherapy, retroperitoneal lymph node dissection was performed, but failed to show any residual viable cells. An ultrasonic study did not reveal any testicular tumors. About 4 years after the previous treatment, he presented with scrotal enlargement and tumor markers such as AFP and HCG β were within normal limit. A right orchiectomy was performed on 23^rd ^July. Pathology showed the resected tumor was a seminoma with CIS (Figure 3A,3B). No recurrence has been seen since the surgery (Figure 1B).

## Conclusions

There are reports that approximately 4% of patients with EGGCT develop a metachronous testicular cancer despite the use of cisplatin-based chemotherapy [7], and the cumulative risk of developing a metachronous testicular cancer 10 years after a diagnosis of EGGCT is 10.3% [8]. However, there is disagreement over whether EGGCT is a primary disease or metastatic from the burned-out primary testicular lesion. Actually, burned-out tumors have been detected in 76% of cases of EGGCT [9]. CIS is also found via biopsy in 31–42% cases [4,10]. Testicular CIS is thought to have resistance to systemic chemotherapies and to develop later to metachronous testicular cancer. In the present case, the EGGCT was a non-seminomatous germ cell tumor including a yolk sac component, whereas the testicular cancer was a seminoma. We believe CIS was present at the time of the treatment of EGGCT and testicular CIS is so primitive that it could differentiate into any type of germ cells. It is also possible that these metachronously developing germ cell tumors developed independently. A testicular biopsy could clarify the relationship between these tumor cells and the expansion of the disease. In the present case no biopsy was done, but an ultrasonic examination ruled out the possibility of testicular CIS. Giwercman et al. [11] emphasized the necessity of histological examinations of the testis upon an evaluation of EGGCT [12-14] and also urged a careful follow-up for patients with EGGCT who do not have simultaneous testicular cancer.

On the other hand, there is an opinion that any patients with retroperitoneal masses should undergo scrotal ultrasound. Comiter et al. [15] showed definite pathological evidence of a burned-out testicular carcinoma in 5 of 6 patients (83%) with presumed extragonadal germ cell tumors and concluded that scrotal ultrasound studies are useful for the evaluation of the palpably normal testes [15]. Kitahara et al. reviewed the incidence of scrotal echogenic leisions with testicular cancers or burned-out tumors of 22 EGGCT patients and found echogenic changes in 17 patients (77.3%) [16]. This means that disease was overlooked by ultrasonic examinations in 22.7% of cases.

In our case, it is possibile that metachronous testicular cancer oriented in testicular CIS, grew from a burned-out tumor, or was independent of the EGGCT. We should have performed testicular biopsies at the time of the diagnosis of EGGCT and reflect the strategies of treatments of EGGCT.

Now we propose a surveillance protocol of EGGCT as Table 1, concerning with following four points.

1. As we mentioned, the overall risk of development a testicular tumor is not so high(4–10.3%).

2. The side effect of CIS therapy (whether irradaition, orchiectomy or chemotherapy) are significant, especially concerning fertility and androgen production.

3. Testicular tumors early detected by adequate surveillance respond well to treatments.

4. Testicular biopsy is not entitled to detect all the CIS.

## Competing interests

The author(s) declare that they have no competing interests.

## Authors' contribution

IK, MU, HY, KN, TT and ND carried out clinical treatments.

TM carried out histopathological studies.

## Pre-publication history

The pre-publication history for this paper can be accessed here:



## Figures and Tables

**Table 1:** Our proposed surveillance protocol of EGGCT is as below.

**Testicular Biopsy**	At the time of the diagnosis		
**Self-check of the testis**	Every months		
**Scrotal ultrasound study**	-3 years	-5 years	-10 years
	every 3 months	every 6 months	every year
**Tumor markers**	-3 years	-5 years	-10 years
	every 3 months	every 6 months	every year
**CT(chest-pelvic)**	-3 years	-5 years	-10 years
	every 3 months	every 6 months	every year
